# Identification of pyroptosis-related gene signature in nonalcoholic steatohepatitis

**DOI:** 10.1038/s41598-024-53599-8

**Published:** 2024-02-07

**Authors:** Fei Mao, E. Wang, Li Fu, Wenhua Fan, Jing Zhou, Guofeng Yan, Tiemin Liu, Yao Li

**Affiliations:** 1 Ministry of Education Key Laboratory of Metabolism and Molecular Medicine, Department of Endocrinology and Metabolism, Zhongshan Hospital, Fudan University, Shanghai, 200032 China; 2 Department of Laboratory Animal Science, School of Medicine, Shanghai Jiao Tong University, Shanghai, 200025 China; 3 Human Phenome Institute, Fudan University, Shanghai, 200032 China; 4 Key Laboratory of Genetic Engineering, School of Life Sciences, Zhongshan Hospital, Fudan University, Shanghai, 200032 China; 5 Shanghai Key Laboratory of Metabolic Remodeling and Health, Institute of Metabolism and Integrative Biology, Fudan University, Shanghai, 200438 China

**Keywords:** Endocrine system and metabolic diseases, Metabolic syndrome

## Abstract

Non-alcoholic fatty liver disease (NAFLD) has emerged as one of the major causes of liver-related morbidity and mortality globally. It ranges from simple steatosis to non-alcoholic steatohepatitis (NASH) characterized by ballooning and hepatic inflammation. In the past few years, pyroptosis has been shown as a type of programmed cell death that triggers inflammation and plays a role in the development of NASH. However, the roles of pyroptosis-related genes (PRGs) in NASH remained unclear. In this study, we studied the expression level of pyroptosis-related genes (PRGs) in NASH and healthy controls, developed a diagnostic model of NASH based on PRGs and explored the pathological mechanisms associated with pyroptosis. We further compared immune status between NASH and healthy controls, analyzed immune status in different subtypes of NASH. We identified altogether twenty PRGs that were differentially expressed between NASH and normal liver tissues. Then, a novel diagnostic model consisting of seven PRGs including *CASP3, ELANE, GZMA, CASP4*, *CASP9*, *IL6* and *TP63* for NASH was constructed with an area under the ROC curve (AUC) of 0.978 (CI 0.965–0.99). Obvious variations in immune status between healthy controls and NASH cases were detected. Subsequently, the consensus clustering method based on differentially expressed PRGs was constructed to divide all NASH cases into two distinct pyroptosis subtypes with different immune and biological characteristics. Pyroptosis-related genes may play an important role in NASH and can provide new insights into the diagnosis and underlying mechanisms of NASH.

## Introduction

The prevalence of nonalcoholic fatty liver disease (NAFLD) is rapidly increasing worldwide due to the global rise in obesity^1^. NAFLD is categorized into two types—“simple” steatosis or nonalcoholic fatty liver (NAFL), and nonalcoholic steatohepatitis (NASH), distinguished by the presence of inflammation and hepatocyte ballooning^2^. If NASH persists, fibrosis occurs and may further progress to cirrhosis and end-stage liver disease^2^. The multiple-hit hypothesis involving plenty of factors has been posited in the pathological mechanism of NASH, of which inflammation plays a critical role^3^. As triglyceride accumulates in the liver, it triggers the inflammatory response and causes immune cells to release cytokines and inflammatory molecules, causing damage to liver cells and the development of NASH^4^. Therefore, controlling inflammation is crucial in the treatment of NASH.

Pyroptosis, a recent described form of programmed cell death, plays an important role in a variety of physiological processes and disease progression^5^. Pyroptosis is characterized by various features, including the activation of caspase family, the formation of pores in the cell membrane mediated by gasdermin proteins, causing rapid cell swelling and rupture, as well as the activation of various cytokines^5,6^. Therefore, inflammatory vesicles, gasdermin protein, and pro-inflammatory cytokines play crucial roles in the process of pyroptosis.

A comprehensive analysis of changes in pyroptosis characteristics in NASH could be a valuable strategy for diagnosing and exploring the pathological mechanisms of NASH. As we know, previous studies were limited to one or two pivotal factors of pyroptosis, due to technical constraints^7–9^. However, disease occurrence and progression involve a series of factors that form a highly synergistic network. Presently, the advancements in high-throughput genomics technology and bioinformatics analysis have enabled researchers to investigate gene expression profiles at a genomic level, leading to novel insights in the interpretation of genomic outcomes, and offering an ideal resource for performing a comprehensive analysis of pyroptosis and immune regulation in NASH^10–12^. Thus, we performed a systematic bioinformatics analysis to determine the expression levels of pyroptosis-related genes between normal and NASH liver tissues, explore the diagnostic value of these genes, and study the correlations between pyroptosis and immune status. In all, our present study indicated that pyroptosis plays a role in NASH development, which may guide the diagnosis and treatment of the disease.

## Results

### Defining of the expression of pyroptosis-related genes (PRGs) in NASH

**Figure 1:**
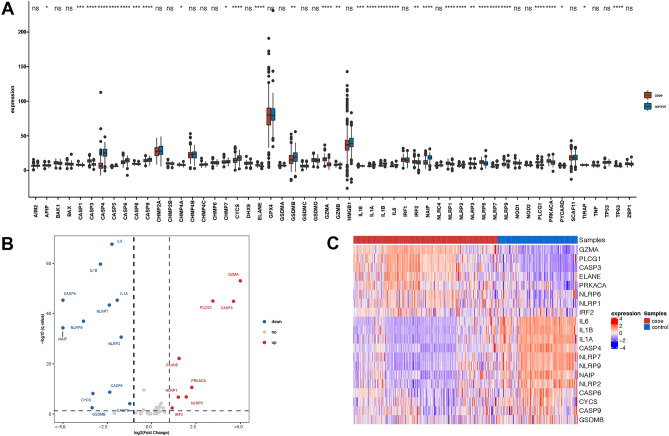
mRNA expression of pyroptosis-related genes in the liver of NASH and normal samples. (**A**) Boxplot of pyroptosis-related genes in the liver of NASH and normal samples. Red color indicates NASH samples (labeled as “case”) and blue color indicates normal samples (labeled as “control”). A fold change of |logFC|> log_2_(1) and FDR of *p* value = 0.05 was set as the screening criterion. The DEGs were signed with * if *p* < 0.05, ** if *p* < 0.01, *** if *p* < 0.001, **** if *p* < 0.0001. (**B**) The volcano plot showed 20 differentially expressed pyroptosis-related genes in the liver of normal and NASH samples. (**C**) The heatmap of 20 differentially expressed pyroptosis-related genes in the liver of normal and NASH samples.

We first acquired a total of 57 pyroptosis-related genes (PRGs) from MsigDB database and published paper. A total of 20 differentially expressed PRGs, either upregulated or downregulated were detected in NASH group (Fig. 1A). More specifically, the expression of *IRF2, NLRP1, NLRP6, PRKACA, PLCG1, ELANE, CASP3,* and *GZMA* was increased, while the expression of *IL6, IL1B, IL1A, CASP4, NLRP7, NLRP9, NLRP2, NAIP, CYCS, CASP6, CASP9* and *GSDMB* was decreased in NASH compared with normal tissues as shown in volcano plot and heatmap (Fig. 1B,C, p < 0.001). Through Pearson correlation analysis in all samples, we detected the strongest relationship between *SCAF11* and *HMGB1* (*R* = 0.72, *p* < 0.001), while as in NASH samples, we found the strongest relationship between *IL6* and *IL1B* (*R* = 0.7, *p* < 0.001), as shown in Supplementary Fig. S1A. These results indicated that expression imbalances of PRGs played important roles in the development of NASH.

Gene locations were further mapped to chromosomes as shown in Supplementary Fig. S1B, mostly of which were located in chromosome 19 and 20. Clinical characteristics of NASH patients from public database were concluded in Table S2.

### Construction of a PRGs‑related diagnostic gene model of NASH

**Figure 2:**
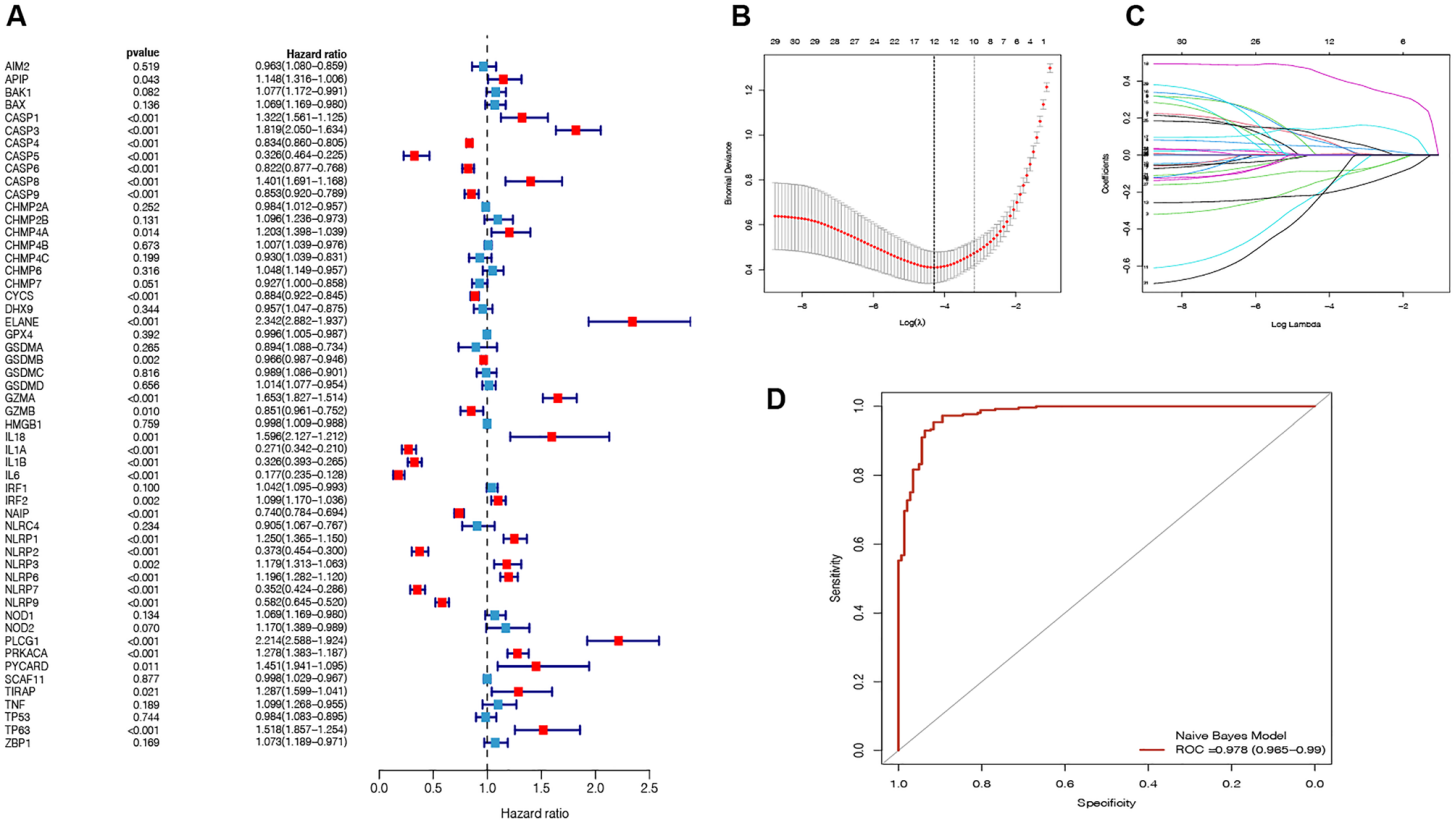
Construction of a diagnostic PRGs model of NASH. (**A**) A forest plot showing the result of univariate logistic regression analysis of PRGs. Hazard ratio and *p*‐value of the constituents involved in univariate logistic regression considering clinical parameters and 31 PRGs in NASH. (**B**) Screening of the optimal parameter (using lambda.1se as the best lambda) at which the vertical lines were drawn. (**C**) LASSO coefficient profiles of the 7 differentially expressed PRGs. (**D**) Receiver operating characteristic (ROC) curve showed the sensitivity and specificity of the diagnostic model by PRGs in NASH.

To further construct a PRGs diagnostic gene model, univariate logistic regression analysis was firstly used for primary screening of PRGs. Altogether 31 genes that met the criteria of *p* < 0.05 were retained for further analysis, and among them, 16 genes were associated with increased risk with hazardous ratio (HRs) > 1, while the other 10 genes were protective genes with HRs < 1 (Fig. 2A). Subsequently, to reduce the number of genes in the model and to solve the multicollinearity problem in regression analysis, we used least absolute shrinkage and selection operator (LASSO) logistic regression to screen feature PRGs. We contained seven genes (*CASP3, ELANE, GZMA*, *TP63, CASP4*, *CASP9* and *IL6)* according to lambda. (Fig. 2B,C). Furthermore, to improve the diagnostic efficiency of PRGs, a novel diagnostic risk score was constructed by multiplying the gene expression. The total risk score was imputed as follows: risk score = (0.278*CASP3 exp.) + (− 0.075*CASP4 exp.) + (− 0.213*CASP9 exp.) + (0.435*ELANE exp.) + (0.301*GZMA exp.) + (− 0.814*IL6 exp.) + (0.54*TP63 exp.) − 2.141. Receiver operating characteristic (ROC) analysis was used to evaluate the diagnostic ability of seven genes, which showed a favorable diagnostic value to NASH, with an AUC of 0.978 (CI 0.965–0.99) (Fig. 2D).

### Human leukocyte antigen (HLA) gene expression profiles, immune cell infiltration profile and immune-related pathway enrichment between control and NASH

**Figure 3:**
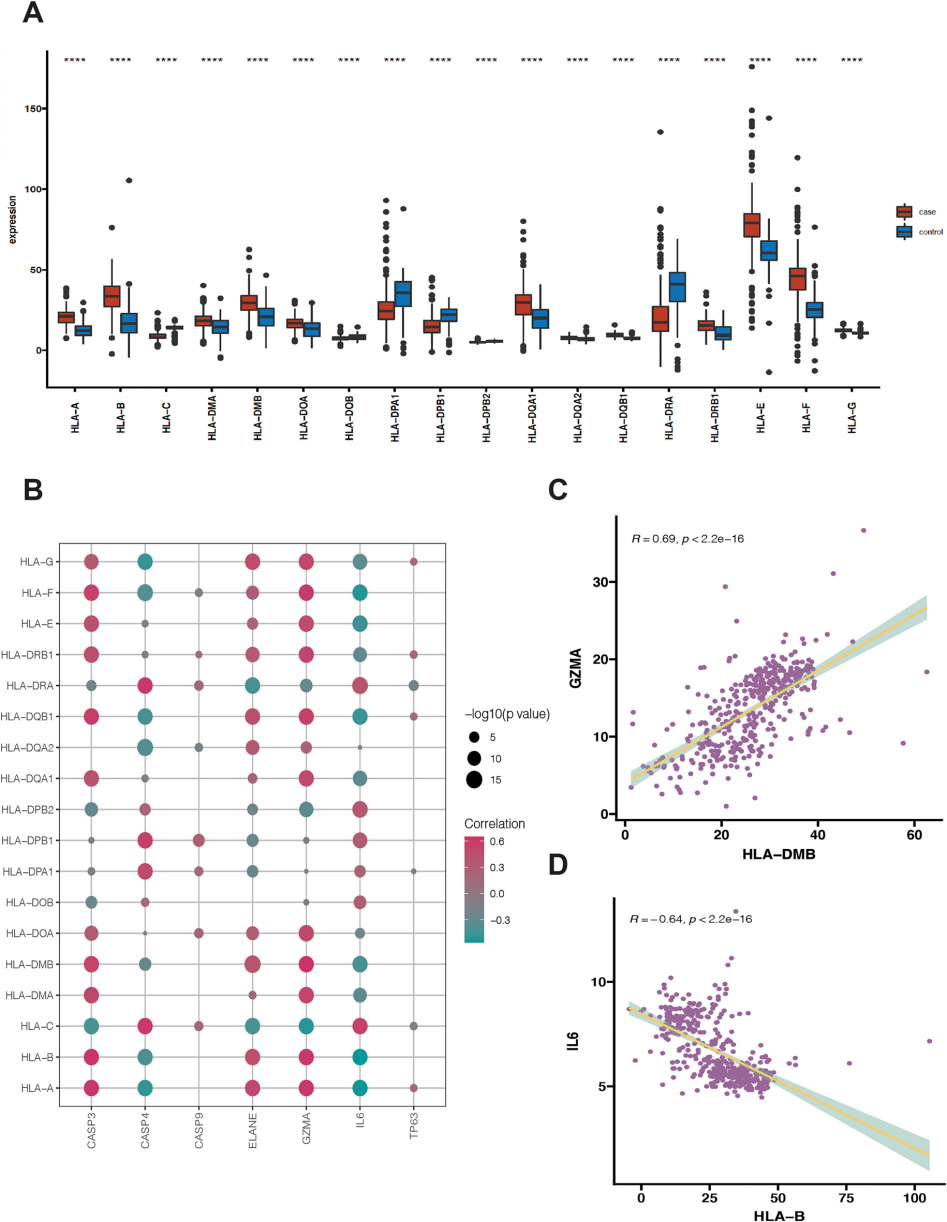
The correlation between HLA related genes and PRGs in the liver of NASH and control samples. (**A**) Boxplot showed differentially expressed HLA related genes in NASH and control samples. Red color indicates NASH samples (labeled as “case”) and green color indicates normal samples (labeled as “control”). (**B**) Dot plot showed Pearson correlation of mRNA expression level between HLA related genes and PRGs including *CASP3, ELANE, GZMA, CASP4*, *CASP9*, *IL6* and *TP63*. (**C**) Dot plot showed Pearson correlations between mRNA expression of *GZMA* and *HLA-DMB*. (**D**) Dot plot showed Pearson correlations between mRNA expression of *IL6* and *HLA-B*. Adjusted *p*-values were showed as: ns, if not significant; * if *p* < 0.05, ** if *p* < 0.01, *** if *p* < 0.001, **** if *p* < 0.0001.

**Figure 4:**
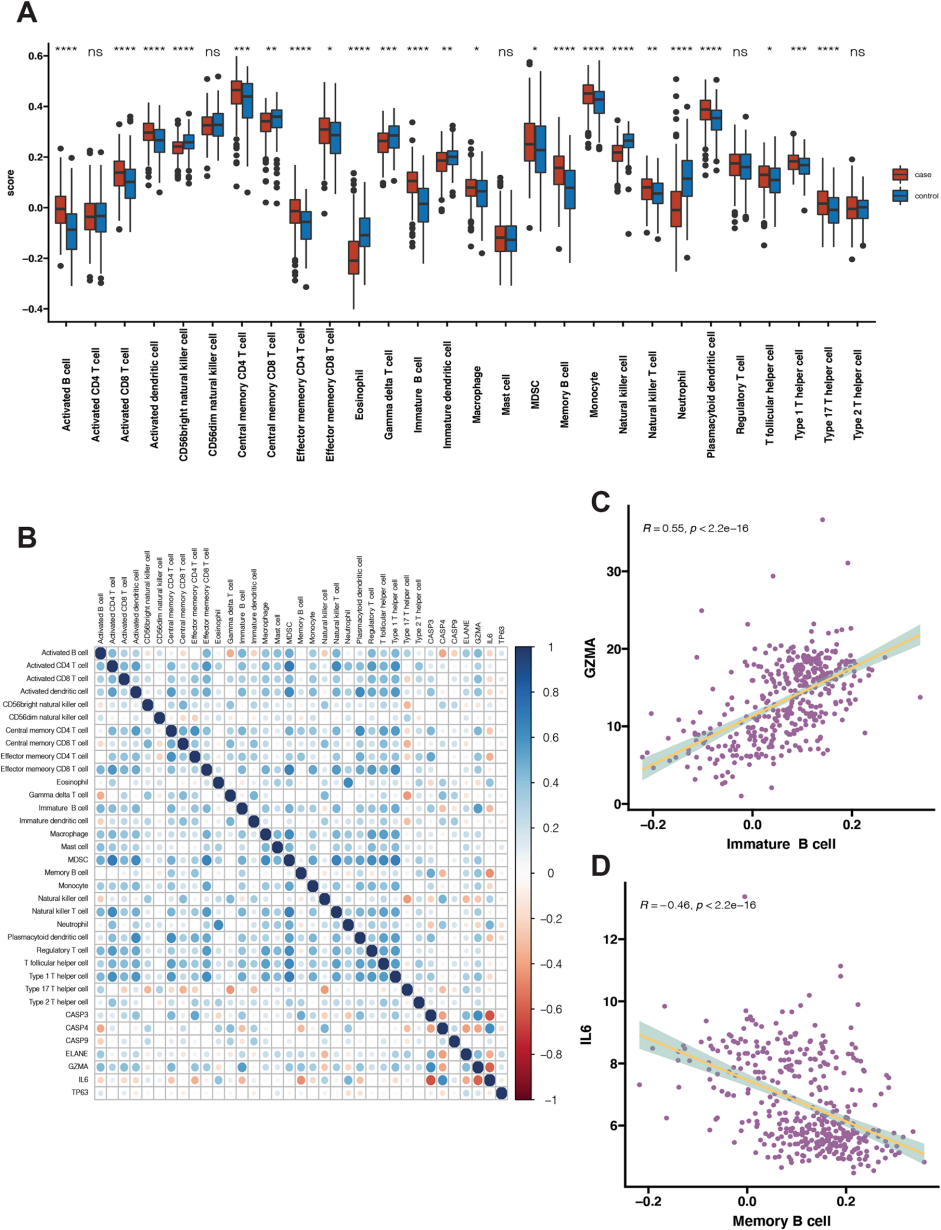
The correlation between infiltrated immune cells and PRGs in the liver of NASH and control samples. (**A**) Boxplot showed differential abundance of infiltrated immune cells in NASH and control samples. Red color indicates NASH samples (labeled as “case”) and green color indicates normal samples (labeled as “control”). (**B**) Dot plot showed Pearson correlation between infiltrated immune cells and 7 PRGs including *CASP3, ELANE, GZMA, CASP4*, *CASP9*, *IL6* and *TP63*. (**C**) Dot plot showed the most positive correlation between immature B cell and *GZMA*. (**D**) Dot plot showed the most negative correlation between memory B cell and *IL6*. Adjusted p-values were showed as: ns, if not significant; * if *p* < 0.05, ** if *p* < 0.01, *** if *p* < 0.001, **** if *p* < 0.0001.

As we know, pyroptosis plays a vital role in the development of the immune microenvironment, thus we also wanted to explore the relationship between pyroptosis and immune response in NASH. The differences in expression of HLA-related genes, immune cell infiltration profile and immune response gene sets between healthy controls and NASH cases were quantified. Firstly, we acquired HLA-related gene datasets and immune biomarkers from previous published paper^13,14^. A total of 18 HLA-related genes were detected in the dataset. These 18 HLA-related genes were all differentially expressed in NASH and normal group as shown in Fig. 3A (*p* < 0.05). Through Pearson correlation analysis of all HLA-related genes with PRGs (As shown in Fig. 3B), we detected the most obvious positive correlation between *GZMA* and *HLA-DMB* (*R* = 0.69, *p* < 0.05) (Fig. 3C) and the most obvious negative correlation between *IL6* and *HLAB* (*R* = − 0.64, *p* < 0.05), respectively, as shown in Fig. 3D. Secondly, ssGSEA was used to calculate the relative abundance of immune cells in each sample. Altogether 23 immune cells showed significant changes in NASH samples. NASH group generally had higher levels of infiltrated immune cells, especially of activated B cell, immature B cell, memory B cell, activated CD8 T cell, activated dendritic cells, macrophage, monocyte than control group (Fig. 4A). These results suggested significantly altered immune cell profiles in NASH samples comparing with healthy controls. Through Pearson correlation analysis of all immune cell markers with PRGs as shown in Fig. 4B, we found that *GZMA* exhibited the most obvious positive correlation with immature B cell (*R* = 0.55, *p* < 0.05), with high expression level in NASH group (Fig. 4C). Meanwhile, *IL6* showed the most obvious negative correlation with memory B cell (*R* = − 0.46, *p* < 0.05), with low expression level in NASH group, as shown in Fig. 4D. Thus, we proposed that these changes immune cells and immune-related pathways played important roles in occurrence and development of NASH.

### Identification of NASH subtypes based on 7 PRGs

**Figure 5:**
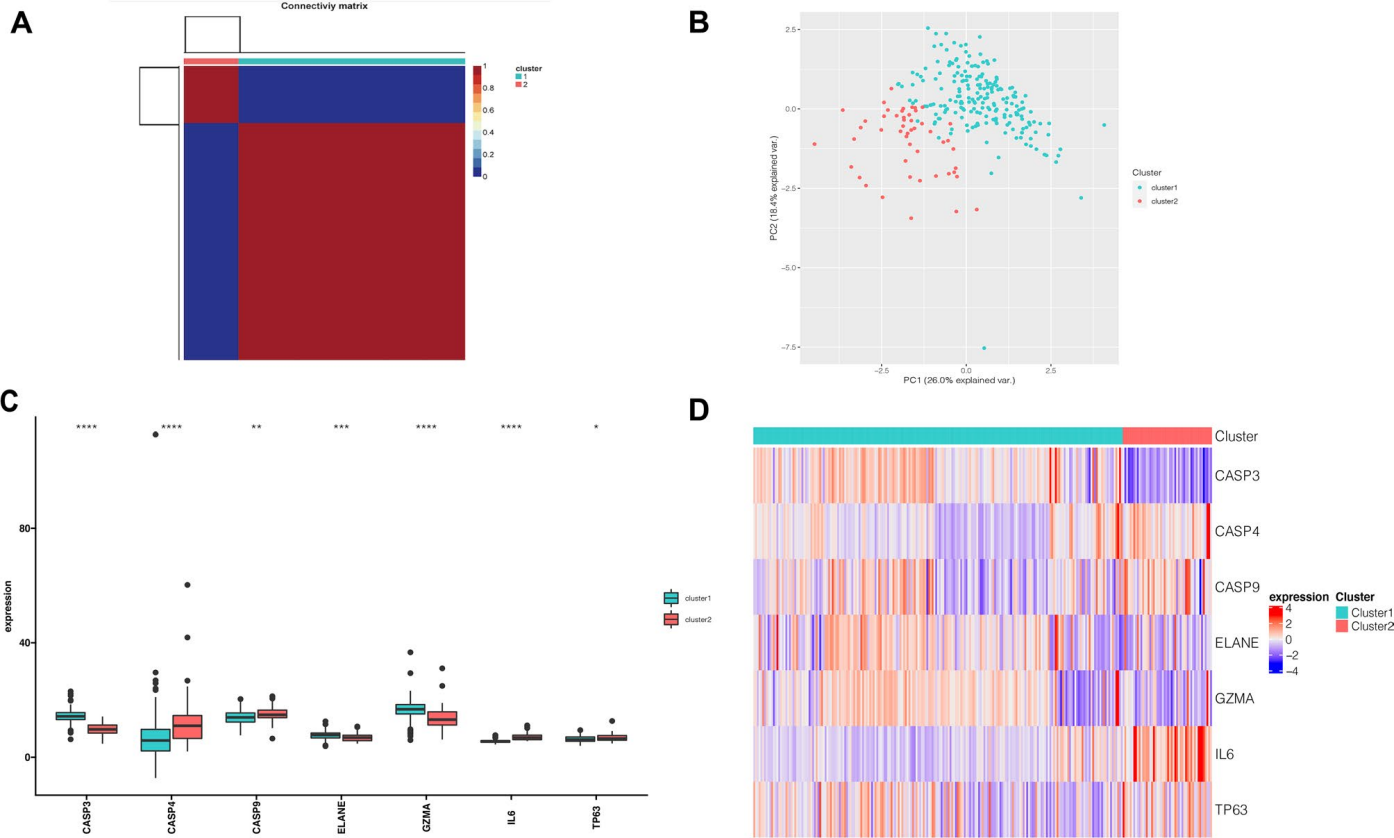
Classification of NASH groups based on consensus clustering of the PRGs. (**A**) A total of 257 NASH patients were grouped into two clusters according to the consensus clustering matrix (k = 2). (**B**) PCA plot showed distinct expression pattern between two subgroups. (**C**,**D**) Violin plot (**C**) and heatmap (**D**) of differentially expressed PRGs in two subclusters of NASH. Red color indicates NASH samples and green color indicates normal samples. Adjusted p-values were showed as: ns, if not significant; * if *p* < 0.05, ** if *p* < 0.01, *** if *p* < 0.001, **** if *p* < 0.0001.

To explore the connections between the expression profiles of 7 PRGs (*CASP3, ELANE, GZMA*, *TP63, CASP4*, *CASP9* and *IL6*) and NASH subtypes, we then performed consensus clustering using data from 257 NASH cases (Fig. 5A). By increasing the clustering variable (k) from 2 to 10, we observed the highest intra-group correlation and lowest intergroup correlation for k = 2, suggesting that the 257 NASH cases could be divided into two clusters according to these PRGs. Altogether 207 NASH samples were included in cluster1 and 50 NASH samples were included in cluster2. PCA analysis indicated these two clusters separated with each other significantly (Fig. 5B). As shown in Fig. 5C,D, the violin plot and heatmap both showed that all those 7 PRGs were differentially expressed between two clusters, in which the expressions of *CASP3, ELANE, GZMA* significantly were increased in cluster1, while the expression of *CASP4*, *CASP9*, *IL6* and *TP63* were decreased in cluster1. However, we found little differences in clinical features between the two clusters (Data not shown).

### Distinct immune and biological characteristics between two clusters

**Figure 6:**
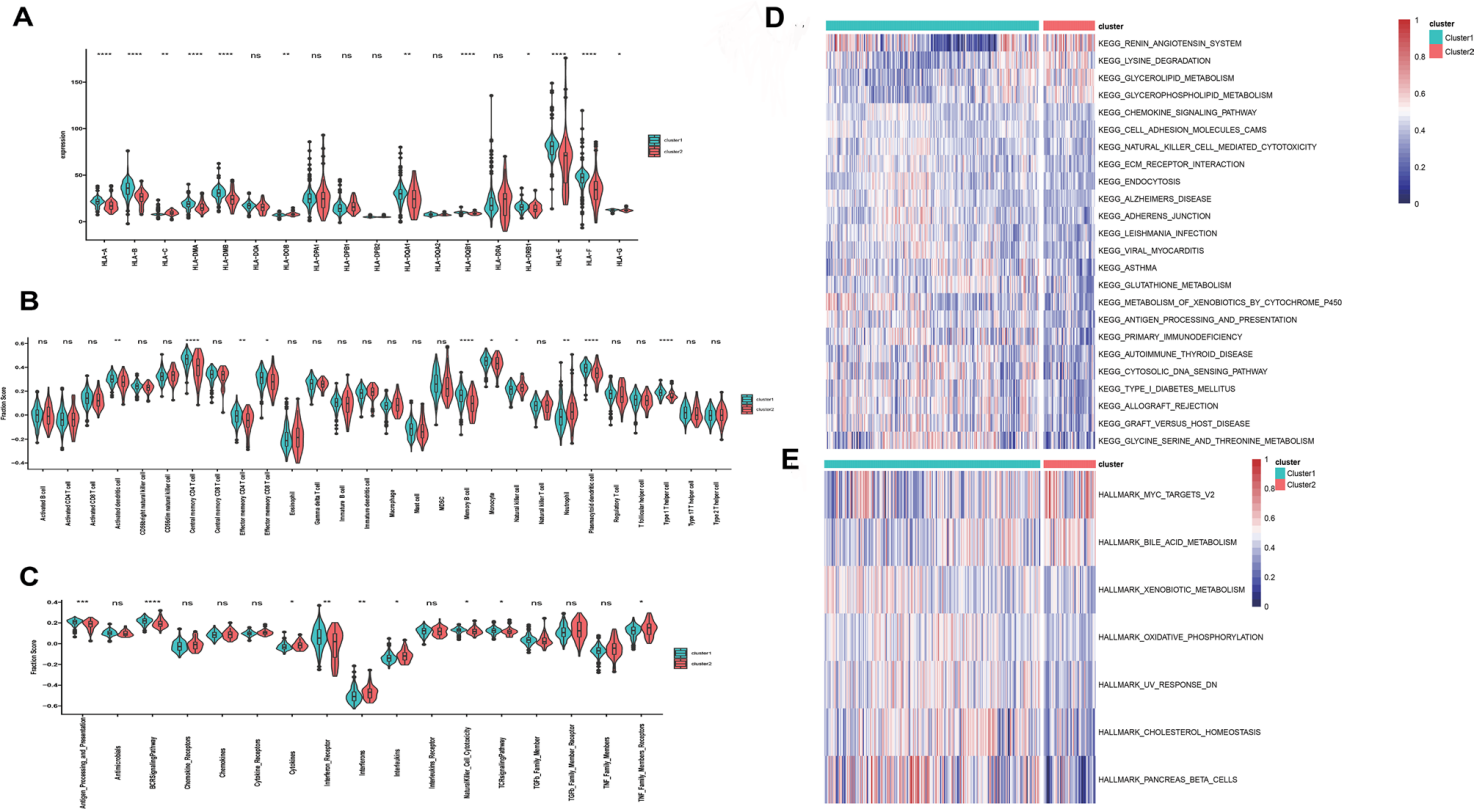
Comparison of immune status in two clusters of NASH. The violin plot showed differential abundance of (**A**) 12 HLA related genes, (**B**) 10 infiltrated immune cells and (**C**) 9 immune-related pathways between two clusters. Green color indicates cluster 1 and red color indicates cluster 2. (**D**,**E**) Heatmap of enriched KEGG analysis (**D**) and mRNA expression level of hallmark genes (**E**) in two clusters. Adjusted *p*-values were showed as: ns, if not significant; * if *p* < 0.05, ** if *p* < 0.01, *** if *p* < 0.001, **** if *p* < 0.0001.

To explore the immune characteristics between two clusters of NASH subtypes, we measured human leukocyte antigen (HLA) gene expression profiles, the immune cells enrichment fractions and immune related pathways. The two clusters revealed different immune characteristics: for example, cluster 1 generally showed high expression of HLA-related genes and infiltration of immune cells, including active dendritic cell, central memory CD4 cell, memory B cell, type-1 T helper cell, as shown in (Fig. 6A,B). Immune pathways also showed differential activity in two clusters (Fig. 6C), of which, 5 immune pathways showed high activity in cluster1, including Antigen processing and presentation, BCR signaling pathway, interferon Receptor, TCR signaling pathway, and Nature killer cell cytotoxicity, while other 4 immune pathways showed high activity in cluster 2.

In addition to immune characteristics, we also explored the biological functions through enrichment scores of Hallmark pathways and Kyoto Encyclopedia of Genes and Genomes (KEGG) pathways by GSVA (Fig. 6D). A total of 24 differential KEGG pathways were obtained, of which, renin angiotensin system, lysine degradation, glycerolipid and glycerophospholipid metabolism pathways showed higher activity in cluster 2 than cluster 1. While other 20 KEGG pathways showed higher activity in cluster 1 than in cluster 2. Through further analysis through hallmark related genes downloaded from MsigDB database, we obtained 7 differentially expressed hallmark related genes including *MYC* targets, bile acid metabolism, xenobiotic metabolism, oxidative phosphorylation, cholesterol homoeostasis, etc. Except for *MYC* target and bile acid metabolism, other hallmark related genes showed higher activity in cluster 1 as shown in Fig. 6E.

## Discussion

NAFLD encompasses a spectrum of liver lesions, from steatosis to cirrhosis, via NASH. The multiple-hit hypothesis involving plenty of factors has been posited in pathological mechanism of NASH^15,16^. Pyroptosis is a type of programmed cell death that is closely related to the inflammatory response^17,18^. While historically much emphasis was placed on apoptosis (i.e., programmed cell death) and necrosis (i.e., non-programmed cell death) in NAFLD^18–20^, however, the role of pyroptosis in NASH remains unclear. Our present study first constructed a diagnosis model for NASH based on PRGs and then explored the role of pyroptosis in NASH.

We firstly studied the mRNA levels of 57 currently known pyroptosis-related genes (PRGs) in NASH and normal tissues and found that a total of 20 PRGs were differentially expressed. Through analysis of PRGs between normal and NASH samples, we found that most of differentially expressed PRGs were caspase family members (*CASP6, CASP9, CASP3, CASP4*) and inflammasome sensors (*NLRP7, NLRP9, NLRP2, NLRP1, NLRP6*). To further assess the diagnostic value of PRGs, we constructed a seven-gene signature via logistic univariate analysis, LASSO regression analysis and logistic multivariate regression analysis and finally screened feature genes and developed a diagnostic model based on seven genes (*CASP3, ELANE, GZMA, CASP4*, *CASP9*, *IL6* and *TP63*). Our present diagnostic model showed the AUC was 0.978 for this model, indicating the high performance for differentiating NASH cases from healthy controls. *CASP3*, known as a classic marker of apoptosis, produces active executors that cleave structural and regulatory proteins in the nucleus and cytoplasm of cells upon activation, thereby regulating cell death. Recent study by Shao et al. showed that activation of *CASP3* can trigger pyroptosis through cleaving GSDME and generating a GSDME-N fragment that perforated membranes^21^. *ELANE* is one of the major serine proteases secreted by neutrophils, and it activates proinflammatory cytokines such as TNF-α, IL-1β, and IL-18, which are known to be pyroptosis promoters^22,23^*. *Kambara et al. proved that GSDMD could be cleaved and activated by *ELANE* and induce neutrophils to undergo pyroptosis^24^. In our study, the expression of *ELANE* was significantly higher while the neutrophil infiltration score was lower in NASH group than in control group, which indicated that *ELANE* might activate pyroptosis in neutrophils. Cytotoxic lymphocyte-mediated immunity relies on granzymes. Shao et al. has reported that *GZMA* could cleave and unleash GSDMD's pore-forming activity, which enables natural killer T cells and cytotoxic T lymphocytes to kill gasdermin B (GSDMB)-positive cells through pyroptosis^25^. In our study, the expression of *GZMA* was higher in NASH group with higher cytotoxic lymphocytes including natural killer T cells. In summary, 3 genes (*CASP3*, *GZMA* and *ELANE*) in the prognostic model were proven to be pyroptosis promoters. However, we also identified down-regulation of *CASP4, CASP9* and *IL6* in NASH patients and related to reduced risk of NASH, while these genes should also be involved in pyroptosis. Till now, pyroptosis has not been fully understood, though shared similarities and crossovers in mechanisms to apoptosis have been found^18^. As disease progresses, multiple ways of cell death may coexist and interact with each other^18^. So, how these genes interact with each other during pyroptosis still remains unclear and requires further investigation.

It is becoming clear that innate immune signaling could modulate the development of certain metabolic diseases, including nonalcoholic fatty liver disease, characterized by chronic, low-grade inflammation due to a dysregulated innate immune signaling^26^. Through further analysis of the immune, we found that most of infiltrated immune cells, including macrophages, activated dendritic cells, immature dendritic cells, activated CD8 T-cells, effector memory CD4 T cells, nature killer T cells, activated B cells, and immature B cells were higher in NASH patients compared with healthy controls. Thus, our results also proved that NASH patients present immune imbalance. As pyroptosis is closely related to the immune response, we further applied correlation analysis between PRGs with immune response and discovered that PRGs were closely associated with the changes of HLA-related genes, immune cells and immune-related pathways in the dataset.

Since we were also interested to explore the immune regulation and search effective anti-inflammatory therapy based on PRGs to target a specific group of NASH patients, we further divided NASH patients into two subtypes (cluster 1 and cluster 2) by using consensus clustering analysis and found different immune status in two subtypes. Patients in cluster 2 presented lower profiles of expression of immune cells, including active dendritic cell, central memory CD4 cell, memory B cell, type-1 T helper cell, indicating lower inflammatory condition. In addition, HLA genes play an important role in immune response and immune therapy. Attractively, most HLA genes were also up-regulated in cluster 1, indicating that patients in cluster 1 might be more sensitive to immune therapy. Five immune pathways showed higher activity in cluster1, including Antigen processing and presentation, BCR signaling pathway, interferon Receptor, TCR signaling pathway, and nature killer cell cytotoxicity, while other four immune pathways showed higher activity in cluster 2. However, unfortunately, the clinical information of NASH patients is limited, the two subtypes did not show any significant differences in clinical characteristics. So, we only conducted a partial study to observe the phenomenon, and further study is needed.

Since very few studies have comprehensively assessed PRGs in NASH, our study preliminarily studied the diagnostic value of these PRGs and systematically analyzed the relationships between PRGs and the immune state in NASH, providing theoretical support for future research. However, our study has several limitations. Firstly, the total sample size was still relatively small (NASH: n = 257; Normal: n = 142) due to a lack of data and we cannot determine the specific role of these PRGs in NASH, which warrants further study. Besides, our study didn't include samples from different NAFLD and NASH stages, which could not reflect the transition from NAFLD to NASH stage. Secondly, this study was based on bioinformatics analysis, and thus many of the results were theoretical. We did not validate the prognostic value of PRGs in the diagnosis of NASH by using validation cohorts. Thirdly, RNA -seq data in GEO database used in our study are all derived from the lysates of whole livers. Though hepatocytes are the major cell type in the liver tissue, other cell types, including Kupffer cells, immune cells and hepatic stellate cells, are also involved in the pathological process of NASH development. Therefore, further in-depth studies are required to deepen our understandings.

In summary, our study provided a molecular model based on PRGs for NASH diagnosis. We also revealed that pyroptosis was related with immune imbalance in NASH.

## Conclusion

Our results provide a potential foundation for future studies of the relationship between pyroptosis and immune environment in NASH. Pyroptosis-related genes may play an important role in NASH and can provide new insights into the diagnosis and underlying mechanisms of NASH.

## Materials and methods

### Data collection

We obtained mRNA-seq datasets from GEO database (GSE48452, GSE83452, GSE89632, GSE164760). Altogether four datasets totally included 257 NASH cases and 142 healthy controls (Table S1). Since clinical information of GSE164760 cannot be accessed, clinical information of 183 NASH cases and 136 control cases were included in Table S2. A total of 57 pyroptosis-related genes (PRGs) were obtained from MsigDB database and published paper and listed in Table S3.

### Identification of differentially expressed genes from GEO database

The expression data in both datasets were normalized to fragment per kilobase million (FPKM) values before comparison. "DMwR2"package and "SVA" package in R studio was used to fill the omitted gaps and remove batch effect. We identified DEGs in all NASH and normal samples using the “Limma” package in R studio. Comparisons between NASHand control group were performed. A fold change of |logFC|> log_2_(1) and FDR of *p* = 0.05 was set as the screening criterion. The DEGs were signed with * if *p* < 0.05, ** if *p* < 0.01, *** if *p* < 0.001, **** if *p* < 0.0001.

### Development of the PRGs diagnostic model

To assess the diagnostic value of the PRGs, we employed univariate logistic regression, analysis, LASSO Cox regression model and multivariate logistic regression analysis to narrow down the candidate genes and to develop the diagnostic model by using R package “glmnet”. Ultimately, seven PRGs and their coefficients were retained, and the penalty parameter (λ) was decided by the minimum criteria. Time dependent receiver operating characteristic (ROC) analysis was applied to evaluate the sensitivity and specificity of the diagnostic model.

### Estimation of immune activity

We analyzed the correlation between seven prognostic PRGs and immune infiltration in NASH samples. Biomarkers of different immune cells and immune pathways were acquired from Msigdb database. A Wilcoxo rank-sum test was used to evaluate the difference in the level of immune cell infiltration in different sample clusters. Single-sample GSEA (ssGSEA) was used to calculate the scores of 28 infiltrating immune cells and the activity of 17 immune-related pathways in different clustering groups using the “GSVA” package of R. We also acquired HLA genes from Msigdb database and compared the expression of the HLA related genes between different clusters.

### Identification of DEGs in distinct pyroptosis regulation subtypes

The consensus clustering algorithm classified NASH patients into two distinct pyroptosis regulation subtypes. We next identified DEGs between two different clusters using the “limma” package. Specifically, gene expression data were normalized using “voom” function and then inputted to the “lmFit” and “eBayes” functions to calculate the differential expressed statistics. The selection criteria were an adjusted *p* value of < 0.05 and Log_2_|FC| of > 1.0.

### Gene set variation analysis and gene ontology annotation

Disease samples were divided into two clusters according to the result from consistent clustering analysis. GSVA KEGG enrichment analyses were conducted using the “ClusterProfiler” package in R studio according to the DEGs (|log_2_FC|≥ 1 and FDR < 0.05) between two clusters. Meanwhile, GSEA was performed in the hallmark gene set “h.all.v7.0.symbols.gmt” to analyze the enriched biological pathways of key genes using GSEA 4.1.0. A *p* value of < 0.05 was considered statistically significant.

### Supplementary Information

Supplementary Figure S1.

Supplementary Table S1.

Supplementary Table S2.

Supplementary Table S3.

## Data Availability

All data generated during this study are included in this published article. The original GEO datasets used for analysis in this study (including GSE48452, GSE83452, GSE89632, and GSE164760) are available in GEO database (https://www.ncbi.nlm.nih.gov/geo/).

## Abbreviations

AUC: Area under the curve
BCR: B cell receptor
DEG: Differentially expressed gene
FDR: False discovery rate
FPKM: Fragment per kilobase million
GSVA: Gene set variation analysis
HLA: Human leukocyte antigen
HR: Hazardous ratio
LASSO: Least absolute shrinkage and selection operator
NAFLD: Non-alcoholic fatty liver disease
NASH: Non-alcoholic steatohepatitis
PRG: Pyroptosis-related gene
ssGSEA: Single-sample gene set enrichment analysis
TCR: T cell receptor
