# Supplementary material for: Identification of pyroptosis-related gene signature in nonalcoholic steatohepatitis
Source: Sci Rep. 2024 Feb 7;14:3175. doi: 10.1038/s41598-024-53599-8 (PMC10850360; doi:10.1038/s41598-024-53599-8)
Supplement: Supplementary file 1 — Supplementary Figure S1. [file 41598_2024_53599_MOESM1_ESM.docx]

**SUPPLEMENTARY INFORMATION**

**
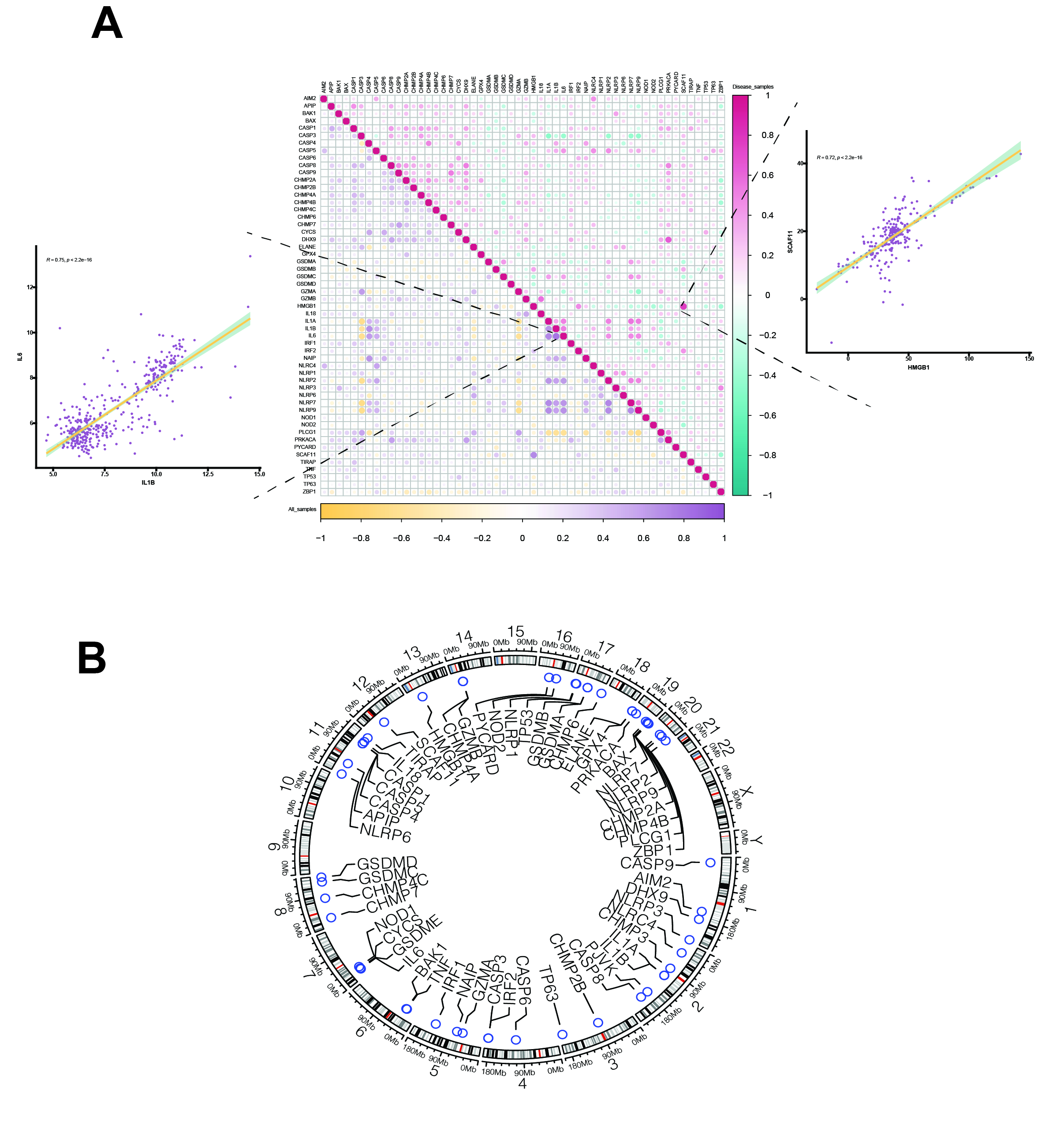
Fig S1.** **Gene pattern and correlation of PRGs detected in the liver of NASH and control samples.** (A) Pearson correlation analysis of PRGs in NASH and control samples. (B) The location of PRGs on 23 chromosomes.
