# Supplementary material for: Identification of pyroptosis-related gene signature in nonalcoholic steatohepatitis
Source: Sci Rep. 2024 Feb 7;14:3175. doi: 10.1038/s41598-024-53599-8 (PMC10850360; doi:10.1038/s41598-024-53599-8)
Supplement: Supplementary file 3 — Supplementary Table S2. [file 41598_2024_53599_MOESM3_ESM.docx]

**Supplementary Table S2. Clinical characteristics of NASH patients from public databases.**

|  | | level | Overall | NASH | Normal |
| --- | --- | --- | --- | --- | --- |
| n | |  | 319 | 183 | 136 |
| Gender (%) | | female | 202 (63.3) | 102 (55.7) | 100 (73.5) |
|  | | male | 117 (36.7) | 81 (44.3) | 36 (26.5) |
| Age (yr) | |  | 43.76 (12.95) | 44.52 (12.46) | 42.75 (13.56) |
| Body mass index (kg/m^2^） | |  | 28.52 (5.10) | 30.24 (5.03) | 25.88 (4.04) |
| HDL-c (mmol/L) | |  | 1.21 (0.31) | 1.14 (0.27) | 1.33 (0.35) |
| LDL-c (mmol/L) | |  | 2.99 (0.91) | 3.04 (0.96) | 2.90 (0.83) |
| Diabetes (%) | |  | 1 (1.6) | 1 (2.6) | 0 (0.0) |
|  | | no | 54 (85.7) | 30 (76.9) | 24 (100.0) |
|  | | yes | 8 (12.7) | 8 (20.5) | 0 (0.0) |
| FBG (mmol/L) | |  | 5.57 (1.71) | 5.94 (2.09) | 5.02 (0.65) |
| Leptin(ug/L） | |  | 22.21 (23.22) | 35.66 (24.35) | 6.84 (6.41) |
| Adiponectin(ug/mL） | |  | 9.50 (7.34) | 6.92 (3.14) | 12.45 (9.56) |
| Fasting insulin level (pmol/L） | |  | 105.24 (154.14) | 127.74 (161.97) | 68.53 (136.61) |
| TC (mmol/L) | |  | 4.87 (1.12) | 4.99 (1.18) | 4.67 (1.01) |
| Fibrosis_stage (%) | | 0 | 31 (56.4) | 21 (53.8) | 10 (62.5) |
|  | | 1 | 14 (25.5) | 8 (20.5) | 6 (37.5) |
|  | | 2 | 2 (3.6) | 2 (5.1) | 0 (0.0) |
|  | | 3 | 4 (7.3) | 4 (10.3) | 0 (0.0) |
| Fibrosis (%) | 0 | | 12 (38.7) | 3 (16.7) | 9 (69.2) |
|  | | 0.5 | 1 (3.2) | 1 (5.6) | 0 (0.0) |
|  | | 1 | 13 (41.9) | 10 (55.6) | 3 (23.1) |
|  | | 2 | 1 (3.2) | 0 (0.0) | 1 (7.7) |
|  | | 3 | 2 (6.5) | 2 (11.1) | 0 (0.0) |
|  | | 3.5 | 1 (3.2) | 1 (5.6) | 0 (0.0) |
|  | | 4 | 1 (3.2) | 1 (5.6) | 0 (0.0) |
|  | | 6 | 3 (9.4) | 3 (16.7) | 0 (0.0) |
|  | | 7 | 1 (3.1) | 1 (5.6) | 0 (0.0) |
| Ballooning_intensity (%) | | 0 | 37 (66.1) | 20 (51.3) | 17 (100.0) |
|  | | 1 | 13 (23.2) | 13 (33.3) | 0 (0.0) |
|  | | 2 | 6 (10.7) | 6 (15.4) | 0 (0.0) |
| Lobular Inflammation (%) | | 0 | 13 (40.6) | 0 (0.0) | 13 (92.9) |
|  | | 1 | 10 (31.2) | 9 (50.0) | 1 (7.1) |
|  | | 2 | 6 (18.8) | 6 (33.3) | 0 (0.0) |
|  | | 3 | 3 (9.4) | 3 (16.7) | 0 (0.0) |
| FBG,Fasting blood glucose; LDL-c, Low density lipoprotein cholesterol; HDL-c, high density lipoprotein cholesterol; TC, total cholesterol; | | | | | |
|  | |  |  |  |  |
|  | |  |  |  |  |
|  | |  |  |  |  |
